# MineProt: a stand-alone server for structural proteome curation

**DOI:** 10.1093/database/baad059

**Published:** 2023-08-10

**Authors:** Yunchi Zhu, Chengda Tong, Zuohan Zhao, Zuhong Lu

**Affiliations:** State Key Laboratory of Digital Medical Engineering, Southeast University, Sipailou No. 2, Nanjing, Jiangsu 210096, China; School of Biological Science and Medical Engineering, Southeast University, Sipailou No. 2, Nanjing, Jiangsu 210096, China; School of Biological Science and Medical Engineering, Southeast University, Sipailou No. 2, Nanjing, Jiangsu 210096, China; School of Biological Science and Medical Engineering, Southeast University, Sipailou No. 2, Nanjing, Jiangsu 210096, China; State Key Laboratory of Digital Medical Engineering, Southeast University, Sipailou No. 2, Nanjing, Jiangsu 210096, China; School of Biological Science and Medical Engineering, Southeast University, Sipailou No. 2, Nanjing, Jiangsu 210096, China

## Abstract

AlphaFold-like systems are rapidly expanding the scale of proteome structuring, and MineProt provides an effective solution for custom curation of these novel high-throughput data. It enables researchers to build their own server in simple steps, run almost out-of-the-box scripts to annotate and curate their proteins, analyze their data via a user-friendly online interface, and utilize plugins to extend the functionality of server. It is expected to support researcher productivity and facilitate data sharing in the new era of structural proteomics.

**Database URL** MineProt is open-source software available at https://github.com/huiwenke/MineProt.

## Introduction

AI-based protein structure prediction systems represented by RoseTTAFold (1) and AlphaFold2 (2) have brought the dawn of the high-throughput era of structural proteomics. With accuracy not inferior to traditional methods, such as X-ray crystallography and cryo-electron microscopy, they have overwhelming advantages in cost, efficiency and ease of operation (1). As of July 2022, AlphaFold Protein Structure Database (3) has open access to over 200 million protein structures, a thousand times the scale of PDB’s 50-year accumulation. Moreover, thanks to contributions from the open-source community, many optimized versions for AlphaFold have been developed, among which ColabFold (4) is undoubtedly a standout. It significantly reduces the resource requirements for protein folding, enabling more researchers to make custom structure predictions and further enlarging the data scale.

When AI systems are rapidly generating structure predictions, problems arise about how to curate these novel high-throughput data. It is difficult for public databases like AlphaFold DB to curate all new data without delays, while it might be hard for most researchers to develop a similar website from scratch to publish their data in a more intuitive way (5). In the history of bioinformatics, a similar situation occurred when next-generation sequencing technology emerged, and it is a stand-alone server called SequenceServer (6) that provides an excellent solution for this issue. Here, we present MineProt, an open-source project to help researchers set up a custom protein server with graphical interface in simple steps. An overview of MineProt features is provided below.

## Results

**Figure 1.**
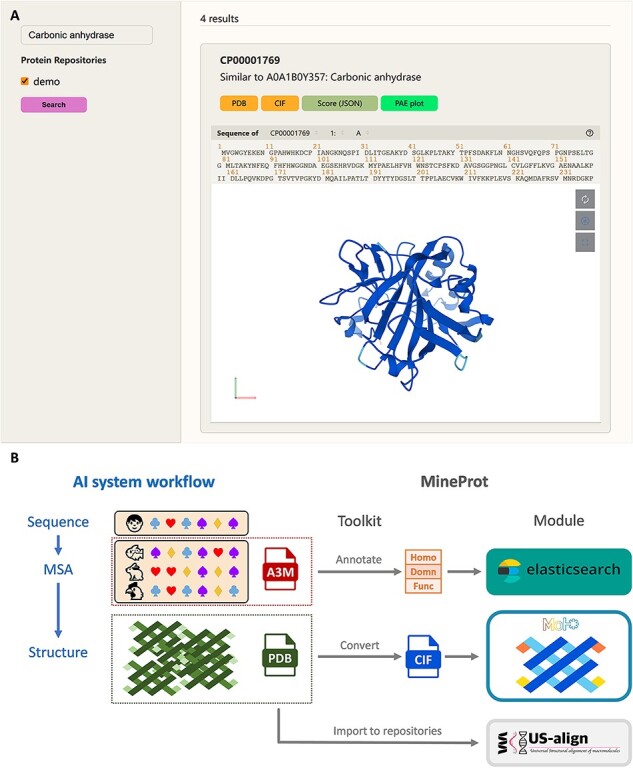
Demonstration of MineProt. (A) The Search Page of MineProt interface. Search scope can be restricted by selecting repositories. The results will be demonstrated in the middle of the page, including annotation, Mol* structure visualization, and links to downloadable file. (B) Basic workflow of MineProt Toolkit. AlphaFold-like systems usually implement a ‘sequence-MSA-structure’ process. MineProt Toolkit uses MSA for homology-based annotation and added them into Elasticsearch indices for full-text searching, meanwhile it converts PDB files to CIF files supporting Mol* visualization. All usable data are finally imported into protein repositories on the server for curation and analysis.

MineProt is designed as server–client architecture. The server supports container deployment including Docker and Podman. It provides a graphical interface for users to curate and analyze their protein structures, which are grouped into different repositories for better management. Users will be able to access the MineProt Search Page (Figure 1A) in a web browser via the server IP address, where all their protein repositories will be listed on the left of the page. One or more repositories can be selected for restricting the search scope and protein information can be retrieved by typing keywords in the search box above. The results will be demonstrated in the middle of the page, including annotation, structure visualization and links to downloadable files. Mol* (7) serves as the structure visualization module. It marks the predicted local distance difference test (pLDDT) scores of each residue in the style of AlphaFold DB, and enables users to zoom, rotate, or take a screenshot.

Users are also enabled to retrieve similar structures via the Salign Page (Video S1) powered by US-align (8). The search scope can be restricted by selecting repositories, entering keywords, or setting maximum root mean square deviation (RMSD). The result page will present Mol* visualization of eligible alignments (TM-score >0.5), where the query structure is gray and the hit structure is in the style of AlphaFold DB, as well as other standard outputs of US-align.

The MineProt interface is reinforced by a Browse Page and an Import Page as well (Figure S1). The Browse Page is used to run over protein data in lists. Under the default configuration, it could demonstrate over 25 000 records for one repository by the aid of efficient caching algorithm, even if they are stored on a low-performance SMR (Shingled Magnetic Recording) hard drive, and the number can easily exceed 75 000 once MineProt is configured to use memory caching. The Import Page is designed to generate command lines for data importing, especially useful when server administrators are new to MineProt scripting.

The client named MineProt Toolkit provides several Python and Shell scripts for the remote management of protein repositories. Its core function can be summarized as transforming results of AI systems to protein repositories compatible with MineProt’s modules, as illustrated in Figure 1B. For instance, if a user employs ColabFold to generate large-scale structure predictions, it only needs one command to invoke MineProt Toolkit to serially unzip result files, convert PDB files to CIF format supporting Mol* visualization, send multiple sequence alignments (MSAs) to UniProt API (9) for homology-based annotations which are transformed into keywords for search engine, and upload all MSAs (.a3m), structures (.pdb and .cif) and model scores (.json) to the target repository. The current version of MineProt Toolkit can directly transform the raw outputs of AlphaFold (up to v2.3.x) and ColabFold (up to v1.5.x) into a fully functional website, while supporting any other data compatible with its standard as well. The CP-8382 dataset, containing 8166 protein structures with computing cost of 4060 GPU hours (5), needs only 92 min for full-flow curation (tested with 10 threads of Intel Core i5-10 400 on a WDC WD20EZAZ-00 L).

In addition, MineProt Toolkit provides plugins for functional extension. For example, given that researchers may expect to search their proteins via BLAST, we have developed a JavaScript plugin linking SequenceServer hits to MineProt Search Page (Video S2). It can either be installed in a web browser via TamperMonkey or directly embedded in the SequenceServer application.

## Discussion

It is expected for MineProt to support researcher productivity and facilitate data sharing in the high-throughput era of structural proteomics. Nevertheless, since MineProt was born upon the arrival of this new era, countless challenges will ambush them both. For example, the compatibility of CIF format emerges to be a problem, for it seems to be too flexible while different software has different processing protocols. The PDB-CIF convertor implemented by MineProt works well for down-stream applications including Mol* and AlphaFill (10), which is an advantage against similar tools such as GEMMI, but it inevitably loses some performance due to its complex processing flow. The definition of a compatible and standard format depends on the exploration and discussion by the entire community, not MineProt or any single application.

Further development of MineProt will continue following the trends in this field. It will support more AI systems, provide more plugins to extend its functionality (handy structure prediction, protein design assist, etc.), update its modules for more efficiency, and become more user-friendly. Such efforts will continue to improve the ability of researchers to analyze and interpret structural proteomics data.

## Methods

### General framework of MineProt server

The MineProt server is implemented in PHP (5.5.9+ with cURL module) and requires one basic web-server platform such as Nginx and Apache. It supports container deployment including Docker and Podman, and a shell script *setup.sh* is provided for one-click installation. The dependencies include Elasticsearch (7.12.1), US-align (20 220 924) and MAXIT (11.100). Elasticsearch is employed as the full-text search engine, where protein annotations serve as keywords in its indices. US-align is the key module for structure alignment, supporting PDB structure searching beyond keyword-searching or BLAST. MAXIT is used for PDB-CIF format converter, which is essential for Mol* visualization. Various APIs (application programming interface) are developed for data management as well as interaction among different modules (see https://github.com/huiwenke/MineProt/wiki/API-manual for details).

### Design of protein repository

MineProt deposits protein-related files in different directories called protein repositories. In the repository, PDB and CIF files storing structure information are required for one protein, while A3M files storing co-evolutionary information and JSON files storing model scores such as pLDDT, predicted template modeling (pTM) and predicted aligned error can also be deposited. Files related to the same protein share the same prefix. Each protein repository has an Elasticsearch index of the same name, which stores descriptions of each of its proteins for searching. Repositories without indices will not be displayed on the MineProt interface, neither searchable nor browsable.

### Workflow of structure alignment service

The Salign Page is designed as a SequenceServer-like interface. It supports PDB–PDB alignment currently. Once receiving query structure and other parameters, MineProt server sends selected repositories and keywords to its search engine to get all candidate structures. After that it creates a background process for US-align calculation between the query structure and candidate structures while returning a job ID to the user. Computing efficiency is affected by hardware, for example, it takes 93.3 s to align 3FM0 from RCSB PDB to 194 WD40-containing proteins from CP-8382 dataset on AMD Ryzen 5 5600 G and over 4 min on Intel Xeon Processor E5-2660 v2. When the job is done, it picks out hit structures with TM-score >0.5 and sort the results by RMSD. Users can access the result page through the returned job ID within 24 h.

### Workflow of MineProt toolkit

MineProt Toolkit is developed in Python (3.6+) with dependencies of three third-party packages, Requests, NumPy and Biopython. Its workflow can be divided into three steps. First, it transforms raw outputs of AI-based protein structure prediction systems into eligible PDB, CIF, A3M and JSON files (*<AI_system>/transform.py*). CIF files are usually generated via MAXIT API of MineProt server. Then, it creates Elasticsearch indices and invokes UniProt API for protein annotation (*import2es.py*). For each protein, homologs in its A3M file are enumerated with multi-threads and sent to UniProt API until registered UniProtKB/UniParc accessions are returned. All available UniProt, InterPro and GO annotations are extracted from the response and added to corresponding Elasticsearch indices. Finally, it uploads all files, which are compressed during transmission, to the target protein repository (*import2repo.py*). Shell scripts integrating all these functions are provided for AlphaFold and ColabFold; meanwhile, a formatting script *ppdb/transform.py* is applicable to most predictions using pTM models.

## Supplementary Material

baad059_Supp

## Data Availability

MineProt is free open-source software (MIT) available at https://github.com/huiwenke/MineProt. Documents are available at https://github.com/huiwenke/MineProt/wiki.
