# Supplementary material for: MineProt: a stand-alone server for structural proteome curation
Source: Database (Oxford). 2023 Aug 10;2023:0. doi: 10.1093/database/baad059 (PMC10414801; doi:10.1093/database/baad059)
Supplement: baad059_Supp [file baad059_supp.zip › suppl_data/Fig_S1.pdf]

Browse demo

⇅ Sort by pLDDT ⇅ Sort by name ⟳ Refresh

Name	Structure	pLDDT	MSA	Annotation
CP00000106	PDB CIF	98.23	A3M	Thioredoxin
CP00004462	PDB CIF	98.10	A3M	retinal dehydrogenase 2-like isoform X1
CP00001235	PDB CIF	98.04	A3M	Glutathione transferase
CP00000602	PDB CIF	97.99	A3M	Peptidyl-prolyl cis-trans isomerase
CP00004464	PDB CIF	97.99	A3M	aldehyde dehydrogenase X, mitochondrial
CP00002762	PDB CIF	97.98	A3M	Glyceraldehyde-3-phosphate dehydrogenase
CP00000604	PDB CIF	97.96	A3M	Peptidyl-prolyl cis-trans isomerase
CP00001477	PDB CIF	97.94	A3M	Type 1 glutamine amidotransferase domain-containing protein
CP00001394	PDB CIF	97.93	A3M	Type 1 glutamine amidotransferase domain-containing protein
CP00001479	PDB CIF	97.92	A3M	Type 1 glutamine amidotransferase domain-containing protein
CP00000098	PDB CIF	97.90	A3M	Uncharacterized protein
CP00002573	PDB CIF	97.89	A3M	Transaldolase
CP00000606	PDB CIF	97.87	A3M	Peptidyl-prolyl cis-trans isomerase
CP00000596	PDB CIF	97.87	A3M	Peptidyl-prolyl cis-trans isomerase
CP00000366	PDB CIF	97.86	A3M	Profilin
CP00000510	PDB CIF	97.84	A3M	Superoxide dismutase [Cu-Zn]

Generate command for importing

**System**	ColabFold
**Repository**	demo
**Naming mode**	0: Use prefix
**Path to data**	/tmp/data
**Path to python3**	/usr/bin/python3
**Path to MineProt scripts**	./toolkit/scripts
**Parameters**	☑ --zip ☑ --amber

Generate

```
cd ./toolkit/scripts

# If your data are raw outputs from colabfold, run:

colabfold/import.sh /tmp/data --repo demo \
--name-mode 0 \
--zip \
--relax \
--python /usr/bin/python3 \
--url http://223.3.59.192

# If your data have been preprocessed by colabfold/transform.py, run:

/usr/bin/python3 import2es.py -n demo -i /tmp/data -a --url http://223.3.59.192/api/es
/usr/bin/python3 import2repo.py -n demo -i /tmp/data --url http://223.3.59.192/api/import2repo/
```
